# Botulinum toxin A injection into the entopeduncular nucleus improves dynamic locomotory parameters in hemiparkinsonian rats

**DOI:** 10.1371/journal.pone.0223450

**Published:** 2019-10-04

**Authors:** Adrianna R. Tsang, Nagalingam Rajakumar, Mandar S. Jog

**Affiliations:** 1 Department of Physiology and Pharmacology, Western University, London, Ontario, Canada; 2 Department of Anatomy and Cell Biology, Western University, London, Ontario, Canada; 3 Department of Clinical Neurological Sciences, London Health Sciences Centre, London, Ontario, Canada; Florey Institute of Neuroscience and Mental Health, The University of Melbourne, AUSTRALIA

## Abstract

Parkinson’s disease is associated with hyperactivity of the subthalamic nucleus (STN), contributing to motor and gait disturbances. Although deep brain stimulation of the STN alleviates certain motor dysfunction, its specific effect on gait abnormalities remains controversial. This study investigated the long-term changes in locomotion following direct infusions of botulinum toxin-A into the globus pallidus internal segment (GPi) to suppress the flow of information from the STN to the GPi in a hemiparkinsonian rat model. Static and dynamic gait parameters were quantified using a CatWalk apparatus. Interestingly, botulinum toxin-A at 0.5 ng significantly reduced only the dynamic gait parameters of hemiparkinsonian rats at 1 week and 1 month post-infusion, while static gait parameters did not change. This study offers new insights into the complexity of basal ganglia in locomotor control and shows the potential of central infusion of botulinum toxin-A as a novel intervention in the study of experimental hemiparkinson’s disease.

## Introduction

Parkinson’s disease (PD) is the second most common neurodegenerative disorder after Alzheimer’s disease. It is estimated that PD affects over 7 million individuals globally and about 1% of the population over 60 years of age [1]. This progressive neurodegenerative disorder is characterized by the loss of dopaminergic neurons in the substantia nigra pars compacta (SNpc), leading to a functional re-organization of the basal ganglia (BG) circuitry including hyperactivity of the subthalamic nucleus (STN) [2,3]. The alterations in the BG network underlie parkinsonian symptoms such as bradykinesia, rigidity, postural instability, and gait disturbances [4].

Classically, gait is defined as the pattern of leg movements used to complete a stride. Each stride comprises of a phase of stand, during which the foot is in contact with the ground and a phase of swing during which the foot is not in contact with the ground. In PD, the automatic and rhythmic pattern of movement required for gait is abnormal leading to short, shuffling steps with freezing episodes, which frequently result in falls that are severely debilitating [5,6]. Patients with PD characteristically present with reduced walking velocity (slowness), shorter step length, shorter stride length and reduced arm swing [7]. While levodopa, a chemical precursor of dopamine, is effective in controlling cardinal motor symptoms of PD, its efficacy on gait abnormalities is questionable [8,9]. Similarly, current literature presents conflicting conclusions on the response of gait to subthalamic deep brain stimulation (STN-DBS). Some studies report an improvement in certain parameters of gait with long term STN-DBS treatment [10–12], whereas others report no benefit or even worsening of gait [13–15]. Thus, it is evident that gait abnormalities and impaired postural stability seen in PD are neither confined to dopaminergic cell loss nor isolated to changes in the BG network. As gait is composed of overlapping static and dynamic parameters, more knowledge about the neurocircuitry involved in specific aspects of gait dysfunction will allow for better selection of a precise intervention for gait improvement in PD patients.

Currently, there are many behavioural tests available to investigate different aspects of gait in rodent models and each are associated with its own strengths and weaknesses. The cylinder test, used to measure forelimb use, akinesia and asymmetry, is limited by the fact that it is manually scored and restricted to only forelimbs [16,17]. Other tests that evaluate forced locomotion, such as the rotarod and treadmill, allow for investigation of more dynamic gait parameters, but do not dissect specific changes in walking patterns [16]. Lastly, footprint tracking, in which an animal walks down a paper after their paws are dipped in ink, only measures static gait [18]. In more recent years, the introduction of the CatWalk gait analysis system has provided for an automated and simultaneous quantification of many static and dynamic aspects of gait during voluntary walking [19]. The CatWalk apparatus consists of a long-enclosed walkway designed to allow animals to traverse freely at their natural speed from one side to the other. The walkway comprises of a glass plate floor illuminated by a green light. As the paws of the animal contact the glass floor, the green light is scattered against a red backdrop. This scattering of light is captured by a high-speed video camera mounted below the glass. Based on the captured data (position, pressure and surface area) of each footprint, the CatWalk software can measure many static and dynamic parameters of gait. Several studies have demonstrated that Catwalk is a reliable tool in assessing gait for rodent models of many neurodegenerative disorders including PD [20–22]. Specifically, this device has been validated for measuring abnormalities in locomotion in the 6-hydroxdopamine (6-OHDA) rodent model of PD [23,24].

The STN is a key glutamatergic nucleus in the basal ganglia network and plays an important role in the facilitation or inhibition of movement [25]. It is well accepted that in PD, the STN is considered overactive due to its increase in glutamatergic outflow to the internal globus pallidus (GPi) [26,27]. Thus, the STN has become a key target site for implementing interventions to treat and manage symptoms of PD [28]. Novel pharmacological therapies that are able to reduce STN neuronal overactivity or impair glutamate transmission at its target structures, such as the GPi, may be highly promising in the treatment of PD. Botulinum toxin type-A (BoNT-A) is a commonly used toxin in medicine, cosmetics and research due to its ability to block the release of acetylcholine from axon terminals [29,30]. BoNT-A has also been demonstrated to preferentially inhibit other excitatory neurotransmitters, including glutamate [31–33]. In the past decade, there has been increasing research to investigate the possibility of direct BoNT-A injections in the brain [34]. Central injections of BoNT-A have been demonstrated to be useful in rodents and promising for a variety of diseases, including PD [35–40]

In the past, we have demonstrated that intrapallidal injection of BoNT-A has the potential to selectively impair overactive glutamate transmission from the STN in the unilateral 6-hydroxydopamine (6-OHDA) rodent model of PD [40]. Accordingly, administration of 0.5 ng of BoNT-A at the entopeduncular nucleus (EPN; rodent equivalent to human GPi) reversed pathological rotational asymmetry and lead to an amelioration of overall gait and walking pattern of hemi-PD animals. Now, in this present study, we quantitatively separate static versus dynamic parameters of gait measured by the CatWalk apparatus and evaluate in detail, changes in these parameters before and after infusion of BoNT-A at the EPN of the 6-OHDA rat model of PD.

## Materials and methods

### Experimental animals

Adult male Sprague-Dawley rats were obtained from Charles River Canada and divided into four experimental groups: (1) sham infused animals receiving vehicle (n = 8), (2) sham infused animals receiving BoNT-A (0.5 ng) (n = 8), (3) 6-OHDA infused animals receiving vehicle (n = 8), and (4) 6-OHDA infused animals receiving BoNT-A (n = 8).

Data collected from these 32 experimental rats was previously published in a prior CatWalk study by this lab and details of animal husbandry can be found in Tsang et al. (2018). All regulatory materials related to the use of BoNT-A in animals, along with all other general procedures associated to the animal studies were approved by the Animal Care Committee at Western University (AUP 2015–087).

### Stereotactic surgery (6-OHDA infusion and BoNT-A Injection)

Consistent with previous animal studies in the lab, stereotactic injection of 6-OHDA into the right medial forebrain bundle (rMFB) was used to create a hemiparkinsonian rodent model [40]. In brief, animals were provided with desipramine hydrochloride (25 mg/kg, IP; Sigma-Aldrich, Oakville, Ontario, Canada) prior to surgery and subsequently, anesthetised with a mixture of ketamine (80 mg/kg, IP) and xylazine (5 mg/kg, IP). 4 μL of 6-OHDA solution (8 μg/rat: 4 μL of 100 mg/50 mL 6-OHDA dissolved in 0.9% saline containing 0.1% ascorbic acid) was infused in the rMFB via a 31G injection cannula attached to a 10 uL Hamilton syringe, at a rate of 1 uL per minute. Correspondingly, sham infused animal groups were administered with an equivalent volume of 0.9% saline with 0.1% ascorbic acid. The stereotactic coordinates with reference to bregma were: AP -1.8 mm, L 2 mm, DV 8.3 mm (Paxinos and Watson, 2009). Success of 6-OHDA infusion was evaluated by the apomorphine-induced rotation test (0.25 mg/kg, SC; Sigma-Aldrich) three weeks after 6-OHDA surgery [40]. Rats demonstrating more than 7 contralateral rotations per minute were considered hemiparkinsonian animals.

Stereotactic injection of BoNT-A was also performed as described previously [40]. 6-OHDA infused and control rats then received BoNT-A injection (List Biological Laboratories, Campbell, California, USA) at the right EPN four weeks after 6-OHDA surgery. Delivery of 0.5 ng of BoNT-A into the EPN was performed by injection of 0.5 uL of BoNT-A solution (0.5 μL of 1 ng/uL BoNT-A dissolved in phosphate buffered saline (PBS) containing 0.1% bovine serum albumin (BSA)). Infusions were performed over 5 minutes via a 31G injection cannula attached to a 10 uL Hamilton syringe. Correspondingly, vehicle animal groups were administered with an equivalent volume of PBS with 0.1% BSA. The stereotactic coordinates with reference to bregma were: AP -2.5 mm, L 3 mm, DV 7.7 mm (Paxinos and Watson, 2009).

### CatWalk gait analysis

The CatWalk XT (Noldus, Wageningen, Netherlands) is an automated quantitative gait analysis system that was used to observe and assess spontaneous gait of voluntarily moving rats. For each gait trail, an animal entered the open end of the walkway and crossed the walkway towards its home cage. A gait trial was considered compliant if the rat traverses the whole platform within 8 seconds and towards the correct direction (i.e. no turns). For each testing session, at least three compliant trials per rat were recorded and non-compliant trials were excluded from the study. One week prior to surgery, naïve rats underwent CatWalk training and baseline testing. Three weeks after 6-OHDA surgery and one week prior to BoNT-A injection, all animals were re-evaluated on the CatWalk to measure changes in gait. CatWalk testing was then repeated at 1 week, 1 month and 3 months after BoNT-A injection for all animal groups.

### TH immunohistochemistry

To visually confirm the success of 6-OHDA infusion, immunostaining for tyrosine hydroxylase (TH) was performed at the striatum and substantia nigra [40]. All experimental animals were perfused with 4% paraformaldehyde and then, brains were postfixed in the perfusion solution and cryoprotected in 25–30% sucrose. Coronal sections 40 μM thick were prepared with a freezing microtome and serial sections were processed immunohistochemically. Staining for TH occurred by incubation of a monoclonal mouse anti-TH antibody (Sigma-Aldrich; #T1299, 1:250), followed by incubation of a biotinylated goat anti-mouse secondary antibody (Vector Laboratories, Burlingame, California, USA, 1:200). Sections were amplified with the avidin-biotin complex method (Vectastain Elite ABC‐HRP Kit; Vector Laboratories) for 1 and detected by the 3,3’-diaminobenzidine method (Sigma-Aldrich). This qualitative verification of 6-OHDA infusion by TH staining has been previously described in detail by our group [40].

### Statistical analysis

Each walkway crossing was analyzed using the CatWalk software. For each CatWalk parameter, a rat’s individual average value or each paw was calculated over three compliant trails and then used to obtain data expressed as group means ± standard errors (mean ± SEM). Statistical analysis was performed by GraphPad Prism 7. All data sets were tested for normality distribution with a D’Agostino and Pearson test prior to statistical analysis. Statistical comparisons of animals before and after BoNT-A injection were performed with a repeated measures one-way ANOVA with a Geisser-Greenhouse correction for the degrees of freedom, followed by a Tukey’s post-hoc test. A critical value for significance of p<0.05 was used throughout the study.

## Results

### Dynamic gait parameters

#### Stand

**Fig 1:**
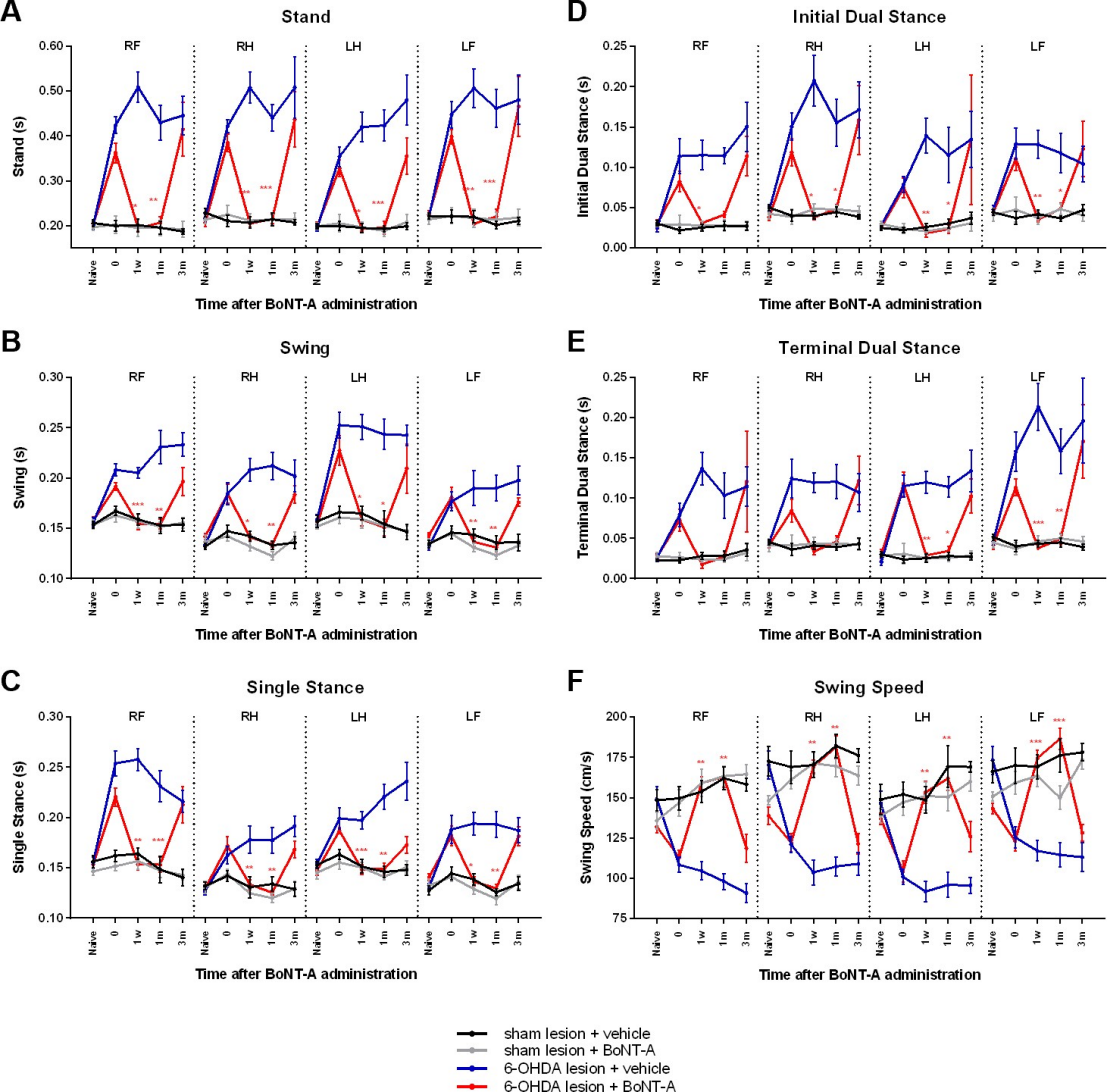
Dynamic gait parameters affected by BoNT-A. Changes in dynamic gait parameters measured by the CatWalk apparatus for each experimental group (n = 8 per group). Paws were measured individually (RF: right front, RH: right hind, LF: left front, and LH: left hind). Experimental timepoints are reflected on the x-axis: naïve rats tested prior to any surgeries, 6-OHDA (or sham infused) animals tested at timepoint 0, and BoNT-A (or sham vehicle) animals tested at 1 week, 1 month or 3 months following BoNT-A surgery. All results are presented as mean ± SEM. Asterisks indicate significant changes compared to timepoint 0 according to repeated measures one-way ANOVA and post-hoc Tukey test. *p<0.5 **p<0.01 ***p<0.001.

Stand is the duration of contact during a step cycle and was measured for each paw (RF: right front, RH: right hind, LF: left front, and LH: left hind). Animals demonstrated a significant increase in stand following 6-OHDA infusion of the MFB (Fig 1A). In fact, the duration of stand approximately doubled from an average of 0.21 (±0.00) seconds to 0.39 (±0.01) seconds across all paws of 6-OHDA infused animals (n = 16). Subsequent administration of 0.5 ng of BoNT-A at the EPN led to significant reduction in stand across all paws of infused animals (red; n = 8). At 1 week following BoNT-A infusion, stand significantly decreased to 0.19 (±0.01) seconds for the RF (F(1.369, 9.586) = 13.64, p = 0.0028, post-hoc p = 0.0021), 0.20 (00B1x0.01) seconds for the RH (F(1.169, 8.181) = 15.38, p = 0.0034, post-hoc p = 0.0002), 0.19 (±0.00) seconds for the LF (F(1.1598, 11.18) = 16.64, p = 0.0007, post-hoc p = 0.0013) and 0.20 (±0.00) seconds for the LH (F(1.147, 8.027) = 15.00, p = 0.0040, post-hoc p < 0.0001). At 1 month following BoNT-A injection, stand remained significantly reduced at 0.21 (±0.01) seconds for the RF (F(1.369, 9.586) = 13.64, p = 0.0028, post-hoc p = 0.0003), 0.21 (±0.01) seconds for the RH (F(1.169, 8.181) = 15.38, p = 0.0034, post-hoc p < 0.0001), 0.20 (±0.01) seconds for the LF (F(1.1598, 11.18) = 16.64, p = 0.0007, post-hoc p = 0.0009) and 0.22 (±0.02) seconds for the LH (F(1.147, 8.027) = 15.00, p = 0.0040, post-hoc p < 0.0001). However, at 3 months following BoNT-A, stand time returned to a longer duration at 0.41 (±0.06) seconds for the RF, 0.44 (±0.06) seconds for the RH, 0.35 (±0.04) seconds for the LF and 0.47 (±0.07) seconds for the LH.

Sham infusion of the MFB did not significantly alter the stand of animals in any of their paws (n = 16). Furthermore, in these sham infused animals, no changes in stand were detected in any of the paws across all timepoints following vehicle injection (black; n = 8) or 0.5 ng of BoNT-A (grey; n = 8) at the EPN. In 6-OHDA infused animals, vehicle injection at the EPN did not reduce the elevated duration of stand at any timepoint (blue; n = 8).

#### Swing duration

Swing duration is the time of no contact between paw and ground during a step cycle. Animals displayed a significant increase in swing duration following 6-OHDA infusion of the MFB (Fig 1B). The time of swing duration across all paws increased from an average of 0.15 (±0.00) seconds in naïve animals to 0.20 (±0.00) seconds in successfully 6-OHDA infused animals (n = 16). Infusion of 0.5 ng of BoNT-A at the EPN led to a significant reduction of swing duration across all paws of infused animals (red; n = 8). At 1 week following BoNT-A injection, swing duration significantly decreased to 0.15 (±0.01) seconds for the RF (F(1.560, 10.92) = 9.226, p = 0.0064, post-hoc p = 0.0001), 0.14 (±0.00) seconds for the RH (F(2.049, 14.34) = 14.50, p = 0.0003, post-hoc p = 0.0133), 0.16 seconds (±0.01) for the LF (F(1.948, 13.64) = 6.704, p = 0.0097, post-hoc p = 0.0258) and 0.14 (±0.00) seconds for the LH (F(1.632, 11.42) = 20.59, p = 0.0003, post-hoc p = 0.0046). At 1 month following BoNT-A infusion, swing duration remained significantly reduced at 0.15 (±0.01) seconds for the RF (F(1.560, 10.92) = 9.226, p = 0.0064, post-hoc p = 0.0025), 0.13 (±0.00) seconds for the RH (F(2.049, 14.34) = 14.50, p = 0.0003, post-hoc p = 0.0052), 0.15 (±0.01) seconds for the LF (F(1.948, 13.64) = 6.704, p = 0.0097, post-hoc p = 0.0154) and 0.13 (±0.00) seconds for the LH (F(1.632, 11.42) = 20.59, p = 0.0003, post-hoc p = 0.0042). However, at 3 months following BoNT-A administration, the longer swing duration of infused animals re-emerged: 0.20 (±0.01) seconds for the RF, 0.18 (±0.01) seconds for the RH, 0.21 (±0.02) seconds for the LF and 0.18 (±0.00) seconds for the LH.

Sham infusion of the MFB had no effect on the swing duration of any of the paws (n = 16). Additionally, in these sham infused animals, vehicle injection (black; n = 8) or 0.5 ng of BoNT-A at the EPN (grey; n = 8) did not significantly alter swing duration in any of the paws across any timepoint. Following vehicle injection at the EPN of 6-OHDA infused animals, swing duration across all paws remained elevated for the following 3 months (blue; n = 8).

#### Single stance

Single stance is the duration of contact for a single paw when the contralateral paw is in swing. Consistent with other CatWalk studies involving hemiparkinsonian rodents, 6-OHDA infusion of the MFB resulted in a significant increase in single stance (Fig 1C). Single stance across all paws increased from an average of 0.14 (±0.00) seconds in naïve animals to 0.20 (±0.00) seconds in 6-OHDA infused animals (n = 16). In these 6-OHDA infused animals, injection of 0.5 ng of BoNT-A at the EPN significantly decreased the single stance across all paws of 6-OHDA infused animals (red; n = 8). Following 1 week of BoNT-A injection, single stance significantly decreased to 0.15 (±0.01) seconds for the RF (F(1.850, 12.95) = 12.19, p = 0.0012, post-hoc p = 0.0065), 0.13 (±0.00) seconds for the RH (F(2.059, 14.41) = 12.92, p = 0.0006, post-hoc p = 0.0063), 0.15 (±0.00) seconds for the LF (F(2.632, 18.42) = 10.21, p = 0.0005, post-hoc p = 0.0005) and 0.14 (±0.00) seconds for the LH (F(2.235, 15.65) = 13.54, p = 0.0003, post-hoc p = 0.0201). After 1 month, the reduced single stance remained significantly lower at 0.15 (±0.01) seconds for the RF (F(1.632, 11.42) = 20.59, p = 0.0003, post-hoc p = 0.0004), 0.13 (±0.00) seconds for the RH (F(2.059, 14.41) = 12.92, p = 0.0006, post-hoc p = 0.0052), 0.15 (±0.01) seconds for the LF (F(2.632, 18.42) = 10.21, p = 0.0005, post-hoc p = 0.0020) and 0.13 (±0.00) seconds for the LH (F(2.235, 15.65) = 13.54, p = 0.0003, post-hoc p = 0.0050). However, 3 months following BoNT-A injection, the single stance of infused animals returned to 0.21 (±0.02) seconds for the RF, 0.17 (±0.01) seconds for the RH, 0.17 (±0.01) seconds for the LF and 0.18 (±0.01) seconds for the LH.

Sham infusion of the MFB did not significantly affect single stance (n = 16). In these sham infused animals, single stance remained unaffected by vehicle injection (black; n = 8) or 0.5 ng of BoNT-A at the EPN (grey; n = 8). In 6-OHDA infused animals, vehicle injection at the EPN had no effect on the increased single stance in any paw (blue; n = 8).

#### Initial dual stance

When a paw ends its swing phase and contacts the ground, the contralateral paw also remains briefly on the ground before it goes into swing. This first phase of bilateral contact is the initial dual stance. Before the first paw enters swing again, the contralateral paw returns to the ground. This second phase of bilateral contact is the terminal dual stance. Following 6-OHDA infusion of the MFB, a significant increase in initial dual stance was observed across all paws (Fig 1D). Initial dual stance across all paws increased from an average of 0.04 (±0.00) seconds in naïve animals to 0.11 (±0.01) seconds in 6-OHDA infused animals (n = 16). Subsequent administration of 0.5 ng of BoNT-A at the EPN significantly decreased the initial dual stance across all paws of 6-OHDA infused animals (red; n = 8). At 1 week following BoNT-A injection, initial dual stance significantly decreased to 0.03 (±0.00) seconds for the RF (F(1.345, 9.418) = 8.499, p = 0.0121, post-hoc p = 0.0337), 0.04 (±0.00) seconds for the RH (F(1.397, 9779) = 7.302, p = 0.0166, post-hoc p = 0.0268), 0.02 (±0.01) seconds for the LF (F(1.046, 7.321) = 1.952, p = 0.2042, post-hoc p = 0.0035) and 0.04 (±0.00) seconds for the LH (F(1.166, 8.161) = 5.502, p = 0.0428, post-hoc p = 0.0058). At 1 month, the shorter initial dual stance remained significantly lower at 0.05 (±0.01) seconds for the RH (F(1.397, 9779) = 7.302, p = 0.0166, post-hoc p = 0.0494), 0.02 (±0.01) seconds for the LF (F(1.046, 7.321) = 1.952, p = 0.2042, post-hoc p = 0.0103) and 0.05 (±0.01) seconds for the LH (F(1.166, 8.161) = 5.502, p = 0.0428, post-hoc p = 0.0179), however was insignificant at 0.04 (±0.00) seconds for the RF. At 3 months following BoNT-A injection, the initial dual stance of infused animals returned to 0.11 (±0.02) seconds for the RF, 0.16 (±0.04) seconds for the RH, 0.13 (±0.08) seconds for the LF and 0.12 (±0.03) seconds for the LH.

Sham infusion of the MFB had no significant effect on initial dual stance (n = 16). Furthermore, in these sham infused control animals, initial dual stance was neither affected by vehicle injection (black; n = 8) or 0.5 ng of BoNT-A at the EPN (grey; n = 8). Vehicle injection at the EPN of 6-OHDA infused animals had no effect on the increased initial dual stance in any paw (blue; n = 8).

#### Terminal dual stance

As described previously, terminal dual stance is the second phase of bilateral contact in a step cycle. Following 6-OHDA infusion of the MFB, a significant increase in terminal dual stance was detected only in the left paws (Fig 1E). Terminal dual stance of the LF increased from 0.03 (±0.00) seconds in naïve animals to 0.11 (±0.01) seconds in 6-OHDA infused animals, and from 0.04 (±0.00) seconds to 0.14 (±0.01) seconds for the LH (n = 16). Although an increase in terminal dual stance was observed in the right paws, it was not significant. Injection of 0.5 ng of BoNT-A at the EPN significantly decreased the terminal dual stance in the left paws of 6-OHDA infused animals (red; n = 8). At 1week post-BoNT-A injection, terminal dual stance significantly decreased to 0.03 (±0.00) seconds for the LF (F(1.502, 10.51) = 11.31, p = 0.0037, post-hoc p = 0.0057) and to 0.04 (±0.00) seconds for the LH (F(1.134, 7.941) = 8.025, p = 0.0201, post-hoc p = 0.0011). At 1 month post-BoNT-A, terminal dual stance remained significantly decreased at 0.03 (±0.01) seconds for the LF (F(1.502, 10.51) = 11.31, p = 0.0037, post-hoc p = 0.0181) and 0.05 seconds for the LH (F(1.134, 7.941) = 8.025, p = 0.0201, post-hoc p = 0.0040). However, at 3 months post-BoNT-A injection, the terminal dual stance of infused animals increased to 0.10 (±0.02) seconds for the LF and 0.17 (±0.05) seconds for the LH.

No significant changes in terminal dual stance were detected following sham infusion of the MFB (n = 16). Similarly, vehicle injection (black; n = 8) or 0.5 ng of BoNT-A at the EPN (grey; n = 8) in these sham infused control animals did not affect the terminal dual stance of the paws. In 6-OHDA infused animals, vehicle injection at the EPN had no effect on the increased terminal dual stance of the left paws (blue; n = 8).

#### Swing speed

The swing speed is the velocity of a paw during the swing phase. Swing speed significantly decreased following 6-OHDA infusion of the MFB (Fig 1F). The average swing speed across all paws reduced from 148.8 (±2.8) cm/second in naïve animals to 114.8 (±2.2) cm/second in 6-OHDA infused animals (n = 16). Infusion of 0.5 ng of BoNT-A at the EPN significantly increased the swing speed across all paws of 6-OHDA infused animals (red; n = 8). At 1 week after BoNT-A administration, swing speed significantly increased to 159.0 (±3.9) cm/second for the RF (F(2.701, 18.90) = 18.47, p < 0.0001, post-hoc p < 0.0001), 169.2 (±4.1) cm/second for the RH (F(2.285, 16.00) = 20.89, p < 0.0001, post-hoc p = 0.0003), 153.4 (±6.7) cm/second for the LF (F(2.551, 17.86) = 10.29, p = 0.0006, post-hoc p = 0.0037) and 174.6 (±4.7) cm/second for the LH (F(2.626, 18.38) = 30.24, p < 0.0001, post-hoc p = 0.0008). At 1 month after BoNT-A injection, swing speed remained significantly elevated at 163.0 (±6.4) cm/second for the RF (F(2.701, 18.90) = 18.47, p < 0.0001, post-hoc p = 0.0022), 181.3 (±7.0) cm/second for the RH (F(2.285, 16.00) = 20.89, p < 0.0001, post-hoc p = 0.0023), 161.9 (±7.5) cm/second for the LF (F(2.551, 17.86) = 10.29, p = 0.0006, post-hoc p = 0.0010) and 186.6 (±6.6) cm/second for the LH (F(2.626, 18.38) = 30.24, p < 0.0001, post-hoc p = 0.0008). However, at 3 months after BoNT-A infusion, infused animals returned to a slower swing speed, 118.7 (±8.8) cm/second for the RF, 121.5 (±6.3) cm/second for the RH, 125.9 (±9.4) cm/second for the LF and 128.3 (±5.3) cm/second for the LH.

Sham infusion of the MFB did not significantly affect the swing speed of animals in any of their paws (n = 16). Additionally, in these control animals, vehicle injection (black; n = 8) or 0.5 ng of BoNT-A at the EPN (grey; n = 8) did not significantly alter swing speed in any of the paws across all experimental timepoints. In 6-OHDA infused animals, vehicle injection at the EPN had no significant effects on swing speed in any paw (blue; n = 8).

### Static gait parameters

#### Stride length

**Fig 2:**
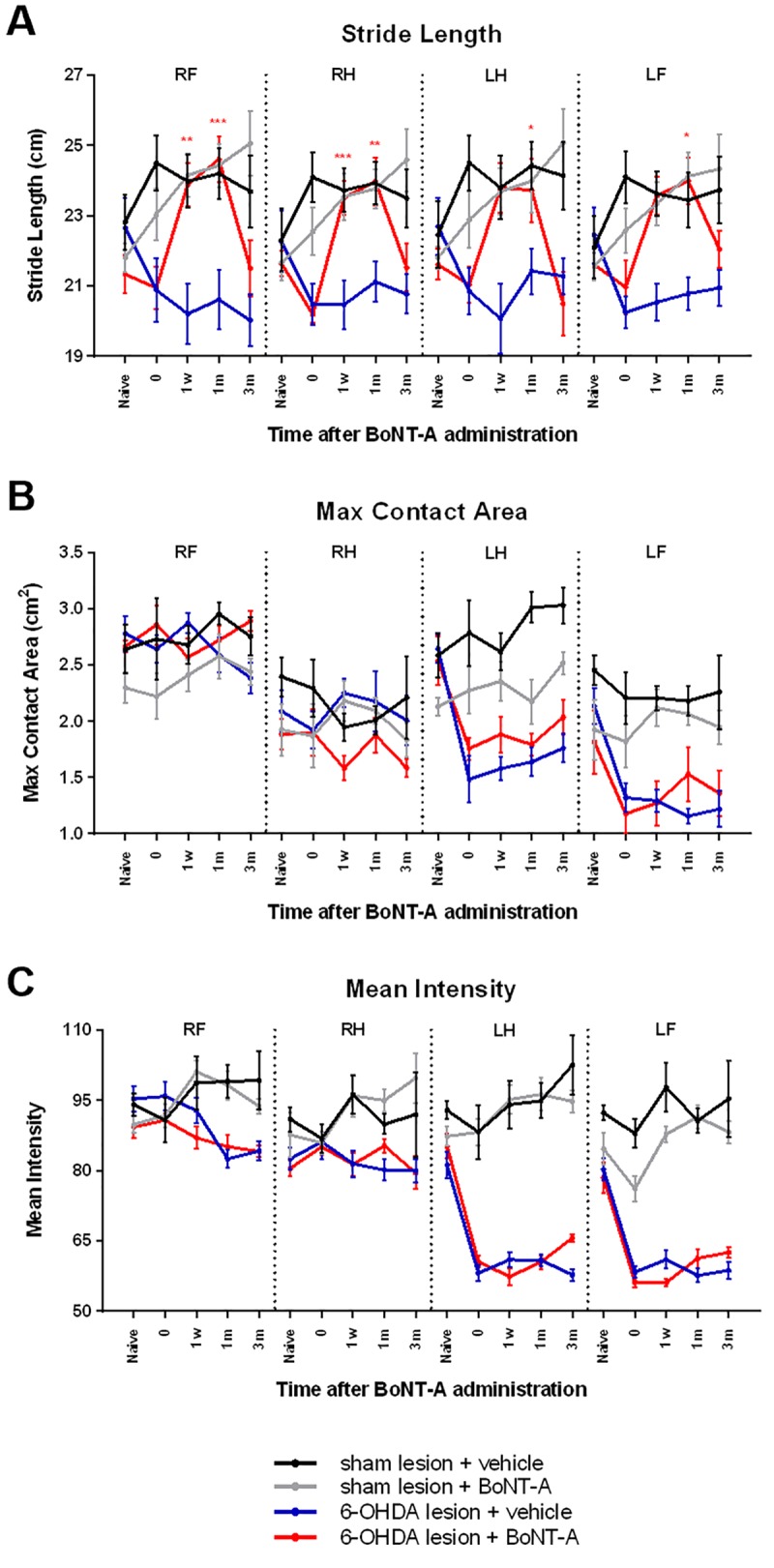
Static gait parameters unaffected by BoNT-A. Changes in static gait parameters measured by the CatWalk apparatus for each experimental group (n = 8 per group). Paws were measured individually (RF: right front, RH: right hind, LF: left front, and LH: left hind). Experimental timepoints are reflected on the x-axis: naïve rats tested prior to any surgeries, 6-OHDA (or sham infused) animals tested at timepoint 0, and BoNT-A (or vehicle) animals tested at 1 week, 1 month or 3 months following BoNT-A surgery. All results are presented as mean ± SEM. Asterisks indicate significant changes compared to timepoint 0 according to repeated measures one-way ANOVA and post-hoc Tukey test. *p<0.5 **p<0.01 ***p<0.001.

The stride length, the distance between two consecutive placements of the same paw, was also measured by the CatWalk apparatus. 6-OHDA infusion of the MFB resulted in a slight reduction in stride length but no significance was detected (Fig 2A). The average stride length across all paws decreased insignificantly from 22.03 (±0.23) cm in naïve animals to 20.70 (±0.21) cm in 6-OHDA infused animals (n = 16). Injection of 0.5 ng of BoNT-A at the EPN significantly increased the stride length 6-OHDA infused animals, but this change was present at different time points for specific paws (red; n = 8). At 1 week following BoNT-A administration, stride length significantly increased to 23.89 (±0.62) cm for the RF (F(2.524, 17.67) = 7.945, p = 0.00021, post-hoc p = 0.0071), 23.50 (±0.48) cm for the RH (F(2.740, 19.18) = 11.35, p = 0.0002, post-hoc p = 0.0003), but no significant changes were detected in either left paws. At 1 month after BoNT-A injection, stride length significantly increased across all paws, to 24.60 (±0.64) cm for the RF (F(2.524, 17.67) = 7.945, p = 0.00021, post-hoc p = 0.0009), 23.97 (±0.69) cm for the RH (F(2.740, 19.18) = 11.35, p = 0.0002, post-hoc p = 0.0085), 23.73 (±0.91) cm for the LF (F(2.789, 19.53) = 6.179, p = 0.0046, post-hoc p = 0.0259) and 23.99 (±0.67) cm for the LH (F(2.672, 18.70) = 5.420, p = 0.0090, post-hoc p = 0.0261). However, at 3 months after BoNT-A infusion, stride length decreased in infused animals to 21.51 (±0.80) cm for the RF, 21.53 (±0.68) cm for the RH, 20.50 (±0.90) cm for the LF and 22.04 (±0.53) cm for the LH.

Stride length was not significantly affected by sham infusion of the MFB (n = 16). Furthermore, following vehicle injection (black; n = 8) or 0.5 ng of BoNT-A (grey; n = 8) at the EPN, no changes in stride length were detected in any of the paws of sham infused animals. Vehicle injection at the EPN of 6-OHDA infused animals did not alter stride length (blue; n = 8).

#### Max contact area

The max contact area of each paw was also measured by the CatWalk apparatus. Consistent with findings from other CatWalk studies involving hemiparkinsonian rodents, 6-OHDA infusion of the MFB resulted in an asymmetric reduction of max contact area in only the left paws (Fig 2B). The max contact area of the LF decreased from 2.59 (±0.12) cm^2^ in naïve animals to 1.62 (±0.12) cm^2^ in 6-OHDA infused animals, and from 1.98 (±0.16) cm^2^ to 1.25 (±0.10) cm^2^ in the LH (n = 16). No significant reduction in max contact area was detected in the right paws of 6-OHDA infused animals. Subsequently, in 6-OHDA infused animals, neither administration of 0.5 ng of BoNT-A (red; n = 8) or vehicle (blue; n = 8) at the EPN had any significant effect on max contact area in either affected (left) or unaffected (right) paws of 6-OHDA infused animals. No changes were detected at any of the tested experimental timepoints.

As expected, max contact area was unaffected by sham infusion of the MFB and subsequent vehicle injection (black; n = 8) or 0.5 ng of BoNT-A at the EPN (grey; n = 8) did not significantly affect the max contact area of any of the paws across all experimental timepoints.

#### Mean intensity

Lastly, the CatWalk apparatus was used to detect the mean intensity of each paw. Similar to the results of max contact area, 6-OHDA infusion of the MFB resulted in an asymmetric reduction of mean intensity in only left paws (Fig 2C). The mean intensity of the LF decreased from 83.18 (±1.95) in naïve animals to 59.35 (±1.05) in 6-OHDA infused animals, and from 79.84 (±1.93) to 57.15 (±0.82) in the LH (n = 16). No significant changes in mean intensity was detected in the right paws of 6-OHDA infused animals. In these hemiparkinsonian animals, infusion of 0.5 ng of BoNT-A at the EPN had no significant effect on mean intensity in either affected (left; n = 8) or unaffected (right) paws (red; n = 8). Likewise, vehicle injection at the EPN had no significant effects on mean intensity of 6-OHDA infused animals (blue; n = 8). No significant changes were observed at any of the tested experimental timepoints.

Sham infusion of the MFB did not significantly affect the mean intensity of animals in any of their paws (n = 16). In these control animals, vehicle injection (black; n = 8) or 0.5 ng of BoNT-A at the EPN (grey; n = 8) did not significantly affect the mean intensity of any of the paws across all experimental timepoints.

#### Summary of gait changes

**Fig 3:**
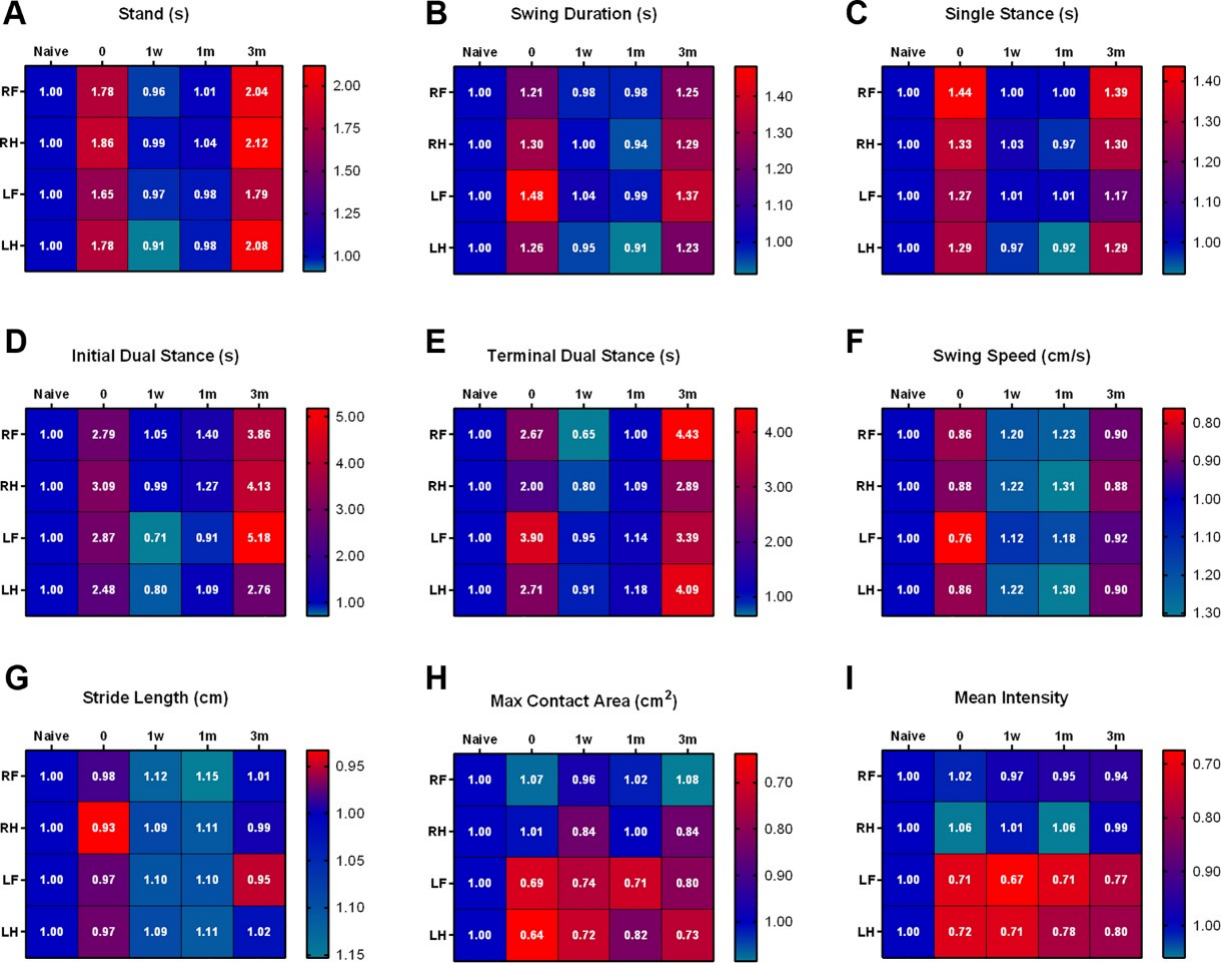
Summary of gait parameters. Summary of gait changes measured by the CatWalk apparatus in 6-OHDA infused rats treated with 0.5 ng of BoNT-A at experimental timepoints (n = 8). For each gait parameter, values represent the fold change compared to the naïve state of each paw. Blue reflects normalcy in the naïve state and red reflects dysfunction in the corresponding colour scale.

A summary of gait changes in 6-ODHA infused rats treated with 0.5 ng of BoNT-A at various timepoints is depicted in Fig 3. Based on the heatmaps, it is evident that dynamic gait parameters (Fig 3A–3F) demonstrate significant improvements at 1 week and 1 month post-BoNT-A infusion. However, at 3 months post-BoNT-A, there is an evident return of gait dysfunction in hemiparkinsonian rats. On the contrary, static gait parameters (Fig 3G–3I) do not follow this same pattern of improvement. Although stride length increases after BoNT-A infusion, the initial impairment caused by 6-OHDA infusion is insignificant. Additionally, the asymmetrical deficits in both max contact area and mean intensity in infused animals do not recover following BoNT-A administration at the EPN.

## Discussion

### Changes in gait following 6-OHDA infusion

Numerous CatWalk studies have reported that following 6-OHDA infusion of the MFB, animals display changes in both dynamic and static gait parameters. Changes in static gait parameters (i.e. features independent of time) following 6-OHDA infusion reported in this study were consistent with findings described by other CatWalk studies [21,24]. Significant reductions in max contact area and mean intensity were only seen in the left paws. This asymmetric presentation in the affected side is due to the unilateral depletion of dopamine in the contralateral hemisphere [16,17]. These gait disturbances in static parameters are most closely linked to an increase in rigidity and altered use of paw surface to support its weight [17,41]. However, not all static gait features displayed this asymmetric presentation and/or were affected by 6-OHDA infusion of the MFB. Although hemi-PD rats in our study displayed a minor reduction in stride length across all limbs, no significance was detected. This is different than data directly reported by another study [24] in which stride length was significantly reduced in PD animals, as well as patients with PD who presented with a decreased stride length [7].

Similarly, the disturbances in dynamic gait parameters (i.e. features dependent on time) following 6-OHDA infusion described in this study were comparable to findings reported by other CatWalk studies [21,24]. Increases in stand, swing duration, single stance and initial dual stance were observed across all paws. These dynamic impairments are thought to model bradykinesia seen clinically in PD patients. A significant reduction in swing speed of infused animals is also thought to be reflective of an increase in muscle rigidity and bradykinesia, as well as a difficulty in the initiation of movements. This difficulty in initiation of movements after 6-OHDA infusion has been reported by various studies that did not utilize the CatWalk apparatus [42–44]. Furthermore, amongst these dynamic gait parameters, an asymmetrical increase in terminal dual stance of the left paws was observed in PD rats. It is postulated that terminal dual stance is the most important parameter in mimicking delays by freezing of gait and postural instability [45,24]. Disturbances in terminal dual stance in the 6-OHDA infused model also reflects a longer double limb support time in PD patients [44]. Overall, this study contributes to the current literature that unilateral 6-OHDA infusion of the MFB induces numerous impairments in both static and dynamic gait parameters, which are assessed by the CatWalk apparatus and can be reproduced with a high degree of fidelity.

### Dynamic gait parameters affected by BoNT-A

Administration of 0.5 ng of BoNT-A at the EPN recovered all impaired dynamic gait parameters in infused animals within a week of infusion, and these improvements persisted for at least a month post-infusion. Reductions in stand and swing duration across all limbs are linked directly to an overall reduction in step cycle duration, which suggest that BoNT-A mediated effects at the BG contribute to an overall reduction in bradykinetic movements during locomotion. Moreover, the improvement in step cycle duration of each limb contributes to the observed increase in average speed. Hence, infused animals with BoNT-A treatment are once again able to traverse the walkway more rapidly because of an increase in step frequency and regularity. Furthermore, the increase in swing speed across all limbs, and especially in the hind paws, suggests that initiation of movements is facilitated by BoNT-A administration. In quadrupeds, slower movements of the hindlimbs is more directly related to disturbances in balance and thus, amelioration of swing speed in the hindlimbs by BoNT-A also likely contributes to the ability of treated animals to maintain a straighter path down the walkway [20]. Lastly, the asymmetric impairment in terminal dual stance was also significantly improved by BoNT-A, which suggests that freezing of gait could be minimized by BoNT-A. Thus, overall, hemiparkinsonian animals treated with BoNT-A can spontaneously walk at a more consistent and rapid pace. These alterations in dynamic gait parameters are postulated to reflect a reduction in bradykinesia, facilitation of motor initiation and minimization of freezing. Amongst dynamic gait parameters, there is also compensation by unaffected paws which contribute to and maintain these improvements.

### Static gait parameters unaffected by BoNT-A

On the contrary, infusion of 0.5 ng of BoNT-A at the EPN failed to ameliorate most dysfunctional static gait parameters of 6-OHDA infused rats. The asymmetric impairments in max contact area and mean intensity in the left paws persisted following BoNT-A administration. Gait disturbances in max contact area and mean intensity are most likely associated to an increase in rigidity and an altered use of the paw surface, and thus unilateral injection of BoNT-A unsuccessfully reduces asymmetric rigidity and/or relieves an imbalanced paw use. A reduction in stride length was observed after 6-OHDA infusion of the MFB, albeit insignificant. However, a significant increase in stride length was detected after administration of BoNT-A in 6-OHDA infused rats. In the right paws, this increase was observed as early as 1 week and in the left paws, it emerged at 1 month. The increase in stride length was similar to the regular stride length of sham infused rats. It is possible that this change in stride length could reflect an altered walking pattern and the ability of animals to take larger and further steps to cross the walkway. Thus, as patients with PD present with smaller shuffling steps, it is postulated that BoNT-A administration could ameliorate a reduced stride length.

### BoNT-A versus other interventions in 6-OHDA Rodents

Although this project is the first to investigate the role of BoNT-A at the EPN of 6-OHDA infused animals with an automated gait analysis tool, other studies have examined the effect of therapies in hemiparkinsonian rats with the CatWalk apparatus. Another study [23] reported that administration of a single dose of L-DOPA in bilaterally infused animals restored most impaired dynamic gait parameters including a reduced swing speed, impaired stance and prolonged step cycle duration. L-DOPA also completely restored the impaired stride length of hemiparkinsonian animals. Although authors did not directly report on other static gait parameters such as max contact area or mean intensity, it was observed that base of support was not affected by L-DOPA. Compared to the improvements induced by BoNT-A at the EPN, there are many parallels between the beneficial effects of these two interventions. Both clearly predominantly affect dynamic gait parameters and have a lesser effect on static gait parameters. With these parallel consequences of L-DOPA and BoNT-A, it is thus likely that the inhibitory effect of BoNT-A acts by modulating the indirect downstream pathway of the dopaminergic system (i.e. STN-EPN connectivity) within the BG. Furthermore, supported by another CatWalk study [23], it is well-established that there is a limited role of L-DOPA therapy for axial symptoms in PD. However, our project examined more gait parameters, including terminal dual stance which best reflects postural instability, and found that BoNT-A improved the asymmetric postural instability of unilaterally infused animals. Thus, by intervening at the level of the EPN and focusing on targeting the glutamatergic system, it is postulated that BoNT-A is exerting a role that extends beyond the dopaminergic motor circuit within the BG. Similarly, another group [21] investigated the rescuing effect of dopaminergic neuron transplantation on gait function of unilaterally 6-OHDA infused animals and found ameliorations in both dynamic and static gait parameters. Following transplantation of dopaminergic neurons derived from embryonic stem cells, 6-OHDA infused rodents demonstrated improvements in max contact area, mean intensity and base of support, as well as swing speed. Furthermore, another group [20] utilized the CatWalk apparatus to examine the effect of high frequency stimulation of the subthalamic nucleus on bilaterally 6-OHDA infused rodents. Bilateral STN electrical stimulation significantly improved overall speed of animals (marked only by a reduction in swing duration) but failed to improve static gait parameters, including max contact area and mean intensity. In fact, it appeared that STN stimulation worsened these static parameters and induced a slowing of forelimb movement (i.e. further reduced swing speed). STN stimulation also failed to have any effect on hindlimb impairments. As authors did not report directly on individual dynamic gait parameters, it is difficult to compare the findings presented in this paper with the results observed in our project. However, as hindlimbs in quadrupeds are mainly involved in the control of balance and postural stability, the findings by this group [20] provide further evidence that electrical stimulation of the STN does not improve deficits in balance and postural instability. This is similar to clinical findings in which bilateral STN stimulation usually fails to restore postural instability and balance impairments in PD patients [15,46]. As our findings suggest that BoNT-A at the EPN does ameliorate many hindlimb deficits of hemiparkinsonian animals and dynamic parameters such as terminal dual stance, there is a clear potential that this intervention could meet some of the shortcomings of DBS. This also indicates that a molecular therapeutic targeting the interaction between the STN-EPN offers different consequences than an electrical intervention. Thus, although the two interventions focus on the same dysregulated sites in PD, the mechanisms behind their effect are unique, but possibly overlapping.

### Time-limited effect of BoNT-A injection

Consistent with other studies that investigated the role of centrally administered BoNT-A in hemiparkinsonian rats [34–40], our results demonstrated that physiological effects induced by BoNT-A injection at the EPN are transient. Prior studies demonstrated a return to dysfunction between 3 and 6 months after BoNT-A injection, depending on the behavioural task [34–40]. In this CatWalk study, the dynamic locomotory parameters of 6-OHDA infused rats returned to levels consistent with pre-BoNT-A injection at 3 months post-BoNT-A injection (i.e. return of hemiparkinsonian impairments). This re-emergence of hemiparkinsonian gait dysfunction at approximately 3 months follows a similar timeline to that of treatment waning and physiological recovery involving direct BoNT-A injections in peripheral targets in a clinical setting [47–49]. As a clinical therapeutic for tremor, the time of peak BoNT-A effect is typically observed at 4 weeks post-injection, with repeat injections occurring every 3–4 months [47–49]. Thus, the central effects of BoNT-A observed in our study reflects a very similar timeline to its well-established peripheral use. Higher doses and/or repeated cycles of BoNT-A injection at the EPN may be promising for a longer and sustained duration of improvement.

### Control systems of gait

Normal gait is regulated by complex, intricate and overlapping loops involving the BG, cerebral cortex and brainstem. BoNT-A administration at the EPN significantly recovered dynamic aspects of gait and thus, it is postulated that dynamic aspects of locomotion could be more heavily influenced by the functional role of the STN and its connectivity to thalamocortical centres via the BG output nuclei, principally the GPi/EPN. When disturbed by 6-OHDA infusion, hemiparkinsonian rodents lose an automaticity and rhythmicity of gait, but time-dependent dynamic gait parameters may be resolved by targeting STN terminals at the EPN by BoNT-A infusion. This is reflected by the improvements in stand and swing duration. These improvements in dynamic locomotory parameters following BoNT-A injection at the EPN could indicate the STN-EPN pathway are preferentially responsible for the disturbance of dynamic features of gait in hemiparkinsonian rats.

In contrast, the control of the static parameters may be different both anatomically and chemically and hence not affected by injection of BoNT-A into the EPN. As static features of gait are more dependent on space and position of limbs, it is postulated the static aspects of gait unaffected by BoNT-A injections at the EPN are linked more closely to alterations in muscle tone and rigidity. The mesencephalic locomotor region (MLR) is long-established to play a key yet not completely understood role in gait [50]. Changes in contralateral MLR activity has been detected and correlated to specific gait deficits in hemiparkinsonian animals tested on the CatWalk apparatus [51]. Along with its connectivity to multiple BG nuclei, the MLR receives fast sensory inputs from the spinal cord to maintain an appropriate position of body segments and baseline level of postural muscle tone. Its anatomical location in the brainstem, between subcortical structures and spinal cord, provides a key role in its control of aspects of gait on balance and posture. The BG control of locomotion and posture predominately utilizes GABAergic output pathways of the substantia nigra pars reticulata (SNpr) to disinhibit the MLR, which sequentially activates reticulospinal tracts [52]. Consequently, BoNT-A infusion at the EPN failed to recover asymmetric static gait impairments seen in hemiparkinsonian animals due to the differential yet complex and intertwined control systems of gait.

## Conclusion

In conclusion, the ability of BoNT-A to significantly improve impaired dynamic gait parameters offers insight to its potential as a novel intervention for PD. This study offers tremendous potential for a variety of translational studies in the future. A similar approach of BoNT-A administration and quantitative gait evaluation can be used in other rodent models of neurodegenerative motor diseases, such as amyotrophic lateral sclerosis and Huntington’s disease. As BoNT-A is commercially available and could be readily paired with established delivery systems, this study serves as a platform for the translation from rodents to primates and ultimately, to humans in the future.

## Supporting information

S1 Dataset sham + vehicle. Data and statistical analysis for sham + vehicle animals.
(RAR)

S2 Dataset sham + BoNT-A. Data and statistical analysis for sham + BoNT-A animals.
(RAR)

S3 Dataset 6-OHDA + vehicle. Data and statistical analysis for 6-OHDA + vehicle animals.
(RAR)

S4 Dataset 6-OHDA + BoNT-A. Data and statistical analysis for 6-OHDA + BoNT-A animals.
(RAR)

S5 Dataset Summary. Data for generation of figures.
(RAR)
